# Generation and Characterization of a *Rdh1‐iCre* Line to Study Uterine Glandular Biology

**DOI:** 10.1002/dvg.70068

**Published:** 2026-07-23

**Authors:** Mana Ohtomo, Shunsuke Takarabe, Takafumi Namiki, Yui Kawata, Ryosuke Kaneko, Manabu Ozawa, Yasuhiro Yamada, Hisashi Mori, Atsuko Kageyama, Maki Kamoshita, Jumpei Terakawa, Junya Ito

**Affiliations:** ^1^ Laboratory of Animal Reproduction, School of Veterinary Medicine Azabu University Sagamihara Japan; ^2^ Graduate School of Veterinary Science Azabu University Sagamihara Japan; ^3^ Medical Genetics Research Center Nara Medical University Kashihara Japan; ^4^ Laboratory of Reproductive Systems Biology, Center for Experimental Medicine and Systems Biology, The Institute of Medical Science The University of Tokyo Minato‐ku Japan; ^5^ Department of Molecular Pathology, Graduate School of Medicine The University of Tokyo Bunkyo‐ku Japan; ^6^ Department of Molecular Neuroscience, Faculty of Medicine University of Toyama Toyama Japan; ^7^ Laboratory of Toxicology, School of Veterinary Medicine Azabu University Sagamihara Japan; ^8^ Center for Human and Animal Symbiosis Science Azabu University Sagamihara Japan

**Keywords:** Cre‐loxP system, mouse, Rdh1, uterine gland, uterus

## Abstract

The uterus is an essential organ for fetal development in most mammals. Uterine glands, highly conserved structures in the mammalian uterus, play critical roles in the establishment and maintenance of pregnancy and have been implicated in the pathogenesis of uterine diseases, including endometrial cancer and endometriosis. Previous studies have shown that Retinol dehydrogenase 1 (*Rdh1*) is specifically expressed in the glandular epithelium (GE) from the onset of gland formation through adulthood. In this study, to develop a GE‐specific Cre driver line, we generated *Rdh1‐iCre* mice by introducing an improved Cre recombinase (*iCre*) into the *Rdh1* locus using the CRISPR/Cas9 system. To evaluate the utility of this model, *Rdh1‐iCre* mice were crossed with *ROSA26‐H2B‐mCherry* reporter mice, and Cre‐dependent reporter expression was analyzed. Robust mCherry fluorescence was observed throughout the uterine glands at 2 weeks after birth, coinciding with the active elongation and branching of the GE. These results demonstrate that the *Rdh1‐iCre* mouse line is a valuable and highly efficient tool for investigating the physiological roles of uterine glands during development and pregnancy, as well as their contribution to the progression of GE‐derived uterine diseases.

## 1 Introduction

The uterus is an essential organ for fetal development in most mammals. It originates from the elongation and fusion of the bilateral Müllerian ducts (paramesonephric ducts), which arise from the invagination of the coelomic epithelium (Kurita 2010; Santana Gonzalez et al. 2021). Although the uterus begins as a simple tubular structure composed of epithelium and mesenchyme during early development, it undergoes maturation into a complex histological structure characterized by the differentiation of the myometrium and uterine glands (Kurita 2010; Machado et al. 2022). Uterine glands are a highly conserved feature of the mammalian uterus; however, their distribution varies by species. In mice and rats, these glands are localized to the antimesometrial side and are absent from the mesometrial side (Vue et al. 2018). In contrast, in ruminants such as cattle and sheep, they are distributed throughout the endometrium except at the caruncles, the specialized sites of implantation (Gray et al. 2001). Uterine glands develop through the invagination and branching of the luminal epithelium (LE), a differentiation process that occurs postnatally in many mammals, including the aforementioned species (Kelleher, DeMayo, et al. 2019).

Uterine glands are indispensable for the establishment and maintenance of pregnancy, processes regulated primarily by progesterone (P4) and 17β‐estradiol (E2). In response to these hormones, the uterine glands secrete cytokines, such as leukemia inhibitory factor (LIF), which are required for successful implantation in mice (Kelleher, Behura, et al. 2019; Namiki et al. 2018; Stewart et al. 1992). Notably, while exogenous LIF can induce implantation in uterine gland‐deficient mice, these animals fail to maintain gestation, suggesting that uterine glands are also critical for post‐implantation pregnancy maintenance (Kelleher et al. 2017). Beyond their reproductive roles, uterine gland abnormalities are central to various pathologies. Endometrial cancer, a malignancy of the uterine epithelium, is thought to arise from the accumulation of mutations in genes such as PTEN within the glandular epithelium (GE) (Daikoku et al. 2008; Liang et al. 2018; Terakawa et al. 2019). Similarly, endometriosis and adenomyosis are estrogen‐dependent inflammatory diseases characterized by the ectopic presence of GE and stroma in extrauterine sites or the myometrium, respectively (Burney and Giudice 2012; Kitawaki et al. 2002; Li et al. 2025). Given the unclear pathogenesis of these conditions, elucidating the developmental mechanisms of the GE is critical for identifying their underlying causes.

In mice, uterine adenogenesis is a postnatal process. Previous research indicates that wingless‐type mouse mammary tumor virus integration site family (WNT)‐catenin beta 1 (CTNNB1) signaling and forkhead box A2 (FOXA2) are vital to the mechanism of uterine gland formation; however, the details of this developmental process remain to be fully elucidated (Dunlap et al. 2011; Kelleher et al. 2018; Kelleher, DeMayo, et al. 2019; Stewart et al. 2013). Additionally, cell‐tracing studies have reported the emergence of progenitor cells responsible for epithelial remodeling during GE differentiation (Seishima et al. 2019; Syed et al. 2020). Research on uterine glands has advanced primarily through the analysis of genetically modified mice, with the Cre‐loxP‐mediated conditional knockout system serving as a critical tool for the spatial and temporal control of gene expression. Representative models include the *progesterone receptor* (*Pgr*)‐*Cre* line (Namiki et al. 2021; Soyal et al. 2005), which expresses Cre recombinase throughout the entire endometrium (epithelium, stroma, and myometrium), and the *Lactotransferrin (Ltf*)*‐iCre* (Daikoku et al. 2014), *Wnt7a‐Cre* (Winuthayanon et al. 2010), and *Small proline rich protein 2F* (*Sprr2f*)*‐Cre* (Contreras et al. 2010) lines, which target the uterine epithelium (both LE and GE). Recently, *Serine protease 29 precursor (Prss29)‐Cre* (Kelleher et al. 2022) and *C‐X‐C motif chemokine ligand 15* (*Cxcl15*)*‐Cre* (Kelleher et al. 2025) lines have been reported as systems that express Cre recombinase specifically within the GE. Given that only a limited number of genes are uniquely expressed in the GE, these GE‐specific Cre lines are indispensable tools for elucidating the specific functions and developmental mechanisms of the uterine glands.

In this study, we generated the *Retinol dehydrogenase 1* (*Rdh1*)*‐iCre* mouse as a novel line for GE‐specific Cre recombinase activity in the uterus. RDH1 is a dehydrogenase that catalyzes the conversion of retinol into the retinoic acid biosynthetic pathway in the liver and adipose tissue (Krois et al. 2019; Zhang et al. 2007). Transcriptional profiling of neonatal and adult LE and GE demonstrated that *Rdh1* is highly expressed exclusively in the GE, beginning at the neonatal stage when gland formation initiates (Filant and Spencer 2013). Furthermore, because *Rdh1* shows no significant expression in the LE during either the neonatal or adult stages (Filant and Spencer 2013), it was identified as a promising candidate for a GE‐specific driver. We generated the *Rdh1‐iCre* line by introducing iCre recombinase downstream of the *Rdh1* locus. In the present study, we demonstrate that the *Rdh1‐iCre* mouse is an exceptionally effective tool for GE‐specific genetic manipulation within the uterus.

## 2 Results

### 2.1 Generation of Rdh1‐iCre Knock‐In Mice

**FIGURE 1:**
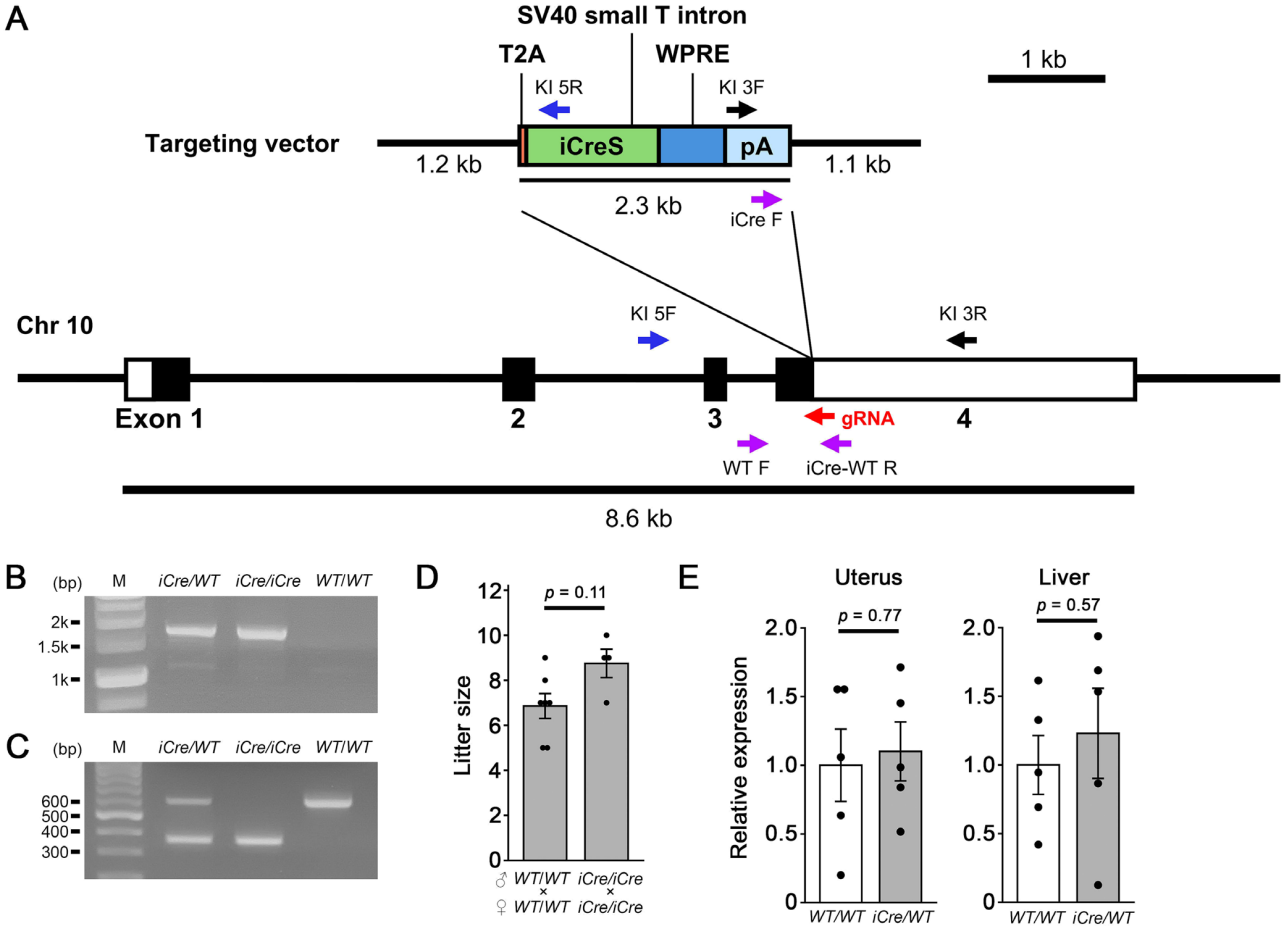
Generation of the *Rdh1‐T2A‐iCre* knock‐in (*Rdh1‐iCre*) mouse. (A) Schematic representation of the targeting vector and its integration into the *Rdh1* locus on Chromosome 10. The targeting vector contains a T2A‐iCreS‐WPRE‐polyA cassette flanked by approximately 1 kb 5′ and 3′ homology arms. The gRNA (red arrow) was designed to target the site immediately upstream of the stop codon for the insertion of the T2A‐iCreS‐WPRE‐polyA sequence. Genotyping primer locations are indicated by blue and black arrows (used in B) and purple arrows (used in C). (B) PCR‐based genotyping using the primers indicated by black arrows (primers KI 3F and KI 3R) in (A). Representative results from mouse tail genomic DNA are shown. Correct knock‐in of the iCre cassette is confirmed by the presence of a 1764 bp amplicon. (C) Genotyping results using the primer set indicated by purple arrows (primers iCre F, WT F, and iCre‐WT R) in (A). The *Rdh1‐iCre* allele yields a 363 bp band, while the wild‐type (WT) allele yields a 605 bp band. (D) Comparison of litter sizes. Data represents the number of offspring from crosses between *Rdh1* ^*WT/WT*^ pairs and homozygous *Rdh1* ^*iCre/iCre*^ pairs. No significant difference was observed between the two groups. (E) Expression levels of *Rdh1* mRNA. Quantitative analysis of *Rdh1* expression in the uterus and liver of *Rdh1* ^*WT/WT*^ (*n* = 5) and *Rdh1* ^*iCre/WT*^ (*n* = 5) mice.

To establish a mouse line that expresses iCre recombinase specifically in the GE, we generated an *Rdh1‐T2A‐iCre* knock‐in line (hereafter referred to as *Rdh1‐iCre*). In this model, the *T2A‐iCre* sequence was inserted in‐frame at the 3′ end of the *Rdh1* coding sequence, immediately upstream of the stop codon (Figure 1A). The insertion of the T2A self‐cleaving peptide sequence allows for the stoichiometric co‐expression of iCre recombinase and the endogenous RDH1 protein from a single polycistronic transcript (Szymczak‐Workman et al. 2012). Heterozygous mice carrying the *Rdh1‐iCre* allele were backcrossed with C57BL/6J mice for at least six generations to standardize the genetic background, after which homozygous *Rdh1‐iCre* mice were produced (Figure 1B,C). Homozygous *Rdh1‐iCre* mice were viable and fertile, showing no reduction in litter size compared to wild‐type (WT) controls (Figure 1D). Furthermore, we confirmed by qPCR that *Rdh1* expression levels in *Rdh1‐iCre* mice were comparable to those in WT mice in both the uterus and liver, indicating that the knock‐in modification did not interfere with endogenous gene regulation (Figure 1E).

### 2.2 GE‐Targeting Cre Recombinase Activity in Rdh1‐iCre Mice

**FIGURE 2:**
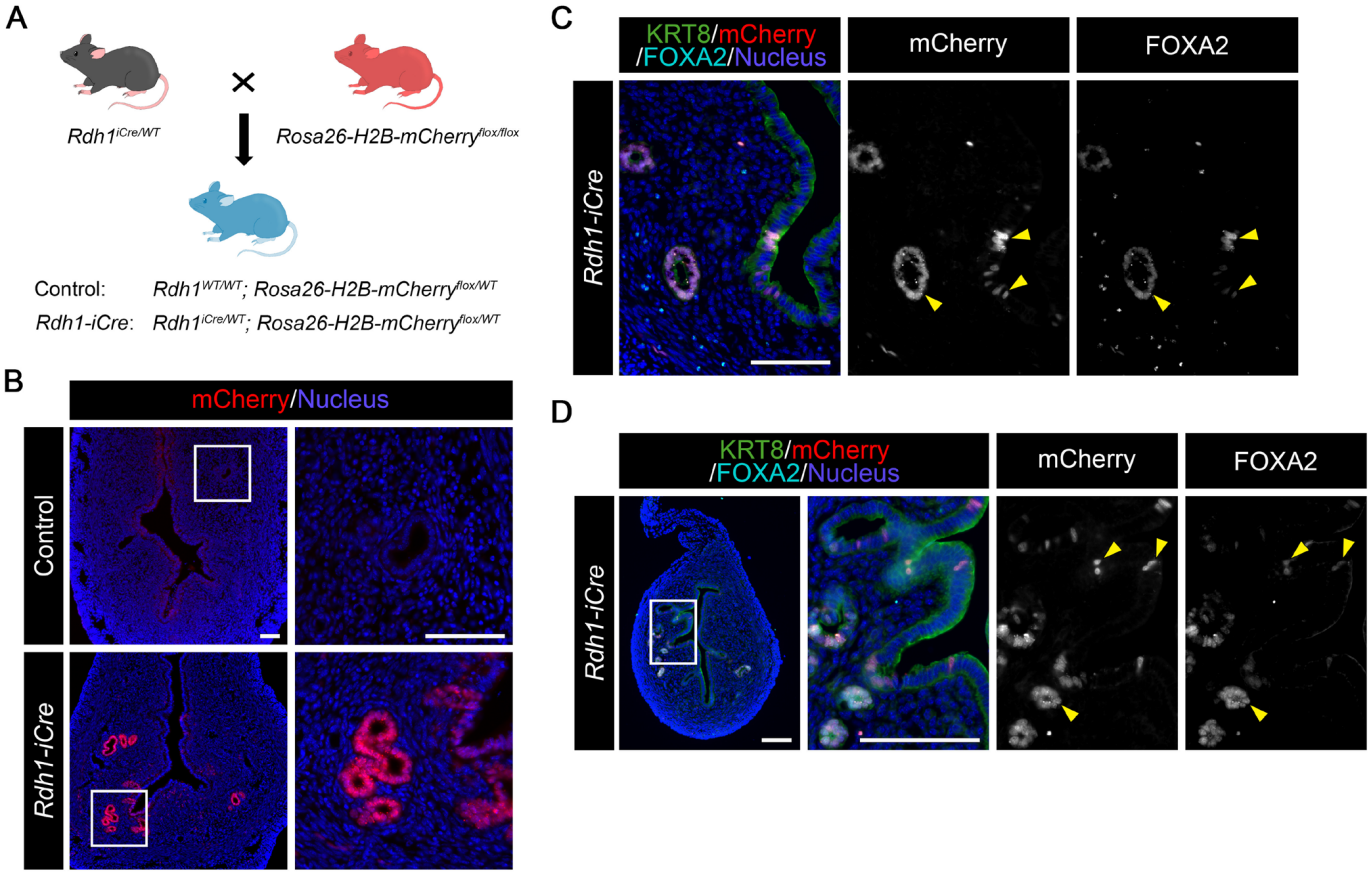
Analysis of *iCre*‐mediated recombination in the uterus of *Rdh1‐iCre* reporter mice. (A) Schematic of the breeding strategy. *Rdh1‐iCre* heterozygous mice (*Rdh1* ^*iCre/WT*^) were crossed with *R26R‐H2B‐mCherry* (*flox/flox*) reporter mice to generate double‐transgenic offspring for lineage labeling. (B) Representative fluorescence images of the adult uterus. Endogenous mCherry signal (red) in uterine sections of *Rdh1* ^*iCre/WT*^; *ROSA26‐H2B‐mCherry* ^*flox/WT*^ mice (≥ 7 weeks of age). Scale bars = 100 μm. (C) Immunofluorescence co‐staining of adult uterine sections. Sections were stained for KRT8 (a general epithelial marker, green) and FOXA2 (a glandular epithelium marker, cyan); nuclei were counterstained with Hoechst (blue). Arrowheads indicate the overlap of the nuclear mCherry signal (red) and FOXA2. Scale bar = 100 μm. (D) Analysis of uterine gland development at 2 weeks of age. Representative fluorescence images showing endogenous mCherry signal (red) and immunofluorescence co‐staining for KRT8 (green) and FOXA2 (cyan). The overlap of mCherry and FOXA2 signals (arrowheads) confirms that Cre‐mediated recombination is active from the onset of adenogenesis. Scale bars = 100 μm.

To evaluate the functional utility of the generated *Rdh1‐iCre* mouse line, heterozygous *Rdh1‐iCre* mice (*Rdh1* ^*iCre/WT*^) were crossed with *ROSA26‐H2B‐mCherry* reporter mice (*ROSA26‐H2B‐mCherry* ^*flox/flox*^) (Abe et al. 2011). In the resulting double‐transgenic offspring, Cre‐mediated excision of the loxP‐flanked stop cassette induces nuclear‐localized mCherry expression in Cre‐expressing cells and their progeny (Figure 2A). Uterine tissues from sexually mature, non‐pregnant females (≥ 7 weeks of age) were collected for analysis. As expected, robust mCherry fluorescence was observed predominantly within the GE (Figure 2B). While mCherry expression was highly concentrated in the GE, a small subset of cells within the LE also exhibited mCherry signal. To further characterize the identity of these mCherry‐positive cells, we performed co‐immunostaining for FOXA2, a transcription factor essential for uterine gland formation and a definitive marker for the GE (Kelleher, DeMayo, et al. 2019). We observed that mCherry expression precisely overlapped with FOXA2‐positive cells within the glandular structures (Figure 2C). Indeed, quantification revealed that nearly all mCherry‐positive cells in the uterus were FOXA2‐positive (98.6% ± 3.0%). Furthermore, analysis of the uterus at postnatal week 2—during active adenogenesis—revealed that invaginating FOXA2‐positive cells were already mCherry‐positive (Figure 2D), while less than 1% of mCherry‐positive cells were FOXA2‐negative. At 1 week of age, a very small number of mCherry‐positive epithelial cells were observed (Figure S1). These data demonstrate that the *Rdh1‐iCre* line enables effective Cre‐mediated recombination starting from the onset of uterine gland differentiation.

### 2.3 Characterization of Cre Recombinase Activity in Extra‐Uterine Tissues

**FIGURE 3:**
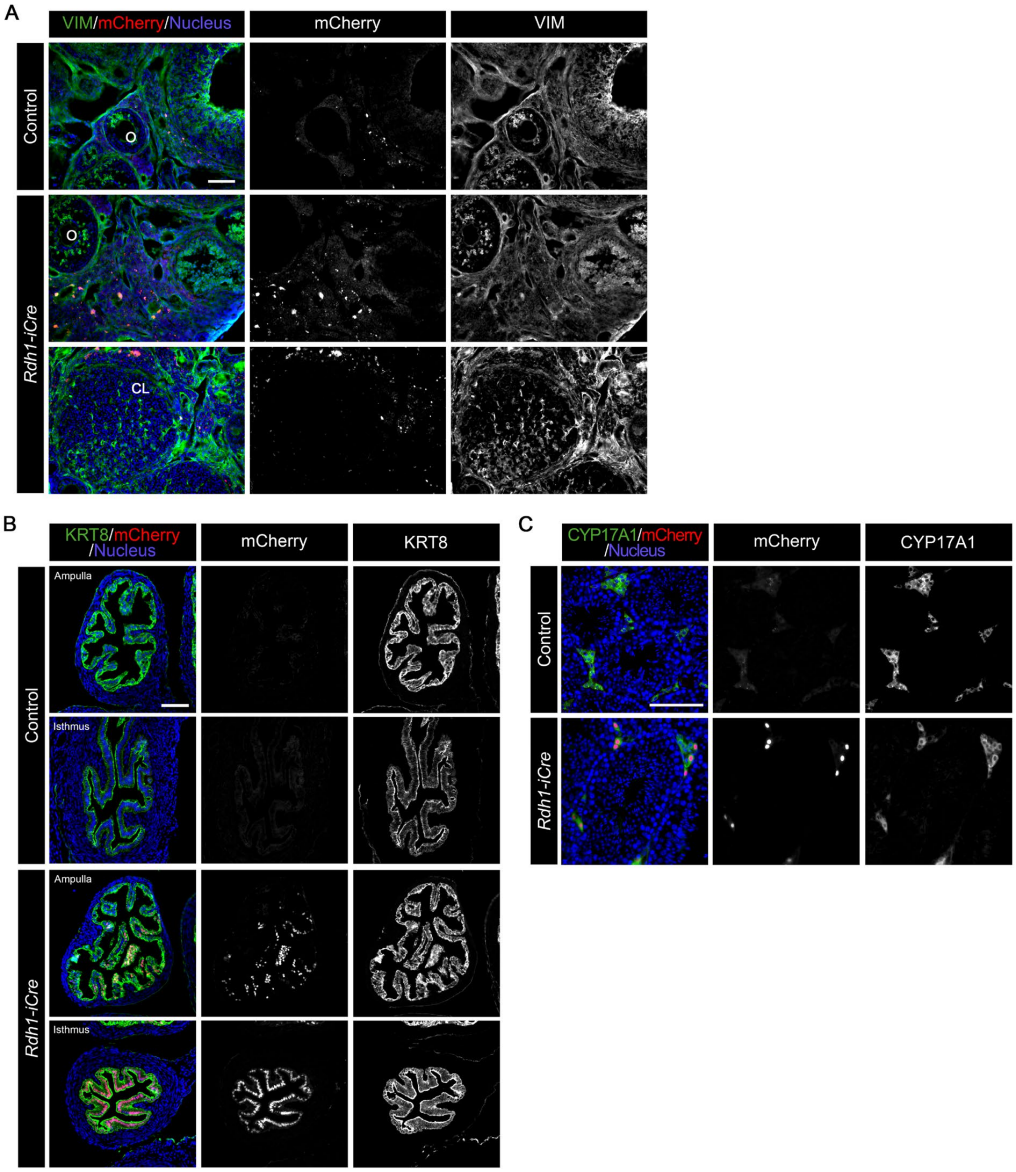
Characterization of *iCre* mediated recombination in extra‐uterine reproductive tissues. Endogenous mCherry expression was analyzed in the reproductive organs of adult *Rdh1* ^*iCre/WT*^; *ROSA26‐H2B‐mCherry* ^*flox/WT*^ mice (≥ 7 weeks of age). Scale bars = 100 μm. (A) Representative fluorescence images of ovarian sections. Endogenous mCherry signal (red) is localized within the ovarian stroma, as demonstrated by co‐immunostaining with the mesenchymal cell marker vimentin (VIM, green). Nuclei were counterstained with Hoechst (blue). O: oocyte; CL: corpus luteum. (B) Representative fluorescence images of oviductal sections. Endogenous mCherry signal (red) is detected in a subset of the oviductal epithelium, including the ampulla and isthmus, as confirmed by co‐immunostaining with the epithelial marker KRT8 (green). (C) Representative fluorescence images of testicular sections. Endogenous mCherry signal (red) is present in the interstitial space. Co‐immunostaining with CYP17A1 (a Leydig marker, green) identifies these mCherry‐positive cells as Leydig cells.

To determine the systemic expression profile of the *Rdh1‐iCre* allele, we examined mCherry reporter expression in the ovaries, oviducts, and male reproductive organs. In the ovary, mCherry expression was predominantly localized to the stromal cells (Figure 3A). Notably, nonspecific red fluorescence was partially observed in ovarian sections. Within the oviduct, mCherry‐positive cells were identified in a subset of the tubal epithelium (Figure 3B). In the testis, mCherry fluorescence was detected in a subpopulation of interstitial Leydig cells, which was further confirmed by co‐staining with the Leydig cell marker CYP17A1 (Figure 3C).

## 3 Discussion

In this study, we generated the *Rdh1‐iCre* mouse as a novel tool for GE‐targeting genetic manipulation. Our results demonstrated that Cre activity is already present throughout the GE by postnatal week 2, shortly after the onset of adenogenesis. This confirms that the *Rdh1‐iCre* line enables genomic recombination from the very beginning of postnatal uterine gland development. Analysis of the *ROSA26‐H2B‐mCherry*; *Rdh1‐iCre* uterus revealed robust mCherry fluorescence in nearly all GE regions, even in heterozygous *Rdh1‐iCre* mice. This indicates that a single copy of the *Rdh1‐iCre* allele is sufficient to drive highly efficient recombination of floxed loci. Notably, mCherry fluorescence was also detected in a subset of cells within the LE. Given that GE cells are known to differentiate from LE cells following the induction of FOXA2 (Kelleher, DeMayo, et al. 2019), and considering that mCherry expression overlapped with FOXA2‐positive cells (Figure 2C,D), the mCherry‐positive LE cells likely represent progenitors destined to become GE. Nearly all mCherry‐positive cells in the uterus at both 2 and 8 weeks of age were FOXA2‐positive. These results suggest that RDH1 expression is closely associated with FOXA2 expression during uterine gland differentiation. Consequently, the *Rdh1‐iCre* mouse serves as an exceptionally powerful tool for investigating the developmental mechanisms and lineage specification of the GE.

Functional analysis of uterine glands in pregnancy and pathologies, such as endometrial cancer, has relied on *Pgr‐Cre* or *Ltf‐iCre* lines (Contreras et al. 2010; Daikoku et al. 2014; Namiki et al. 2021; Soyal et al. 2005; Winuthayanon et al. 2010). However, because these lines also target the LE, isolating gland‐specific functions remains challenging. While *Foxa2‐Cre* mice have been reported (Uetzmann et al. 2008), FOXA2 is expressed in the node, notochord, floor plate, and definitive endoderm during early development; thus, using this line can lead to embryonic lethality or developmental defects, limiting its utility as a GE‐specific driver. More recently, the *Prss29‐Cre* line was reported as a GE‐specific model (Kelleher et al. 2022). However, target gene deletion in *Prss29‐Cre* mice requires the animal to undergo at least one pregnancy. This constraint makes it impossible to analyze gene function during adenogenesis, pre‐pubertal stages, or in nulliparous disease models (Kelleher et al. 2022). In contrast, our *Rdh1‐iCre* mouse achieved nearly 100% recombination in GE cells starting from the adenogenic stage. While the recently reported *Cxcl15‐Cre* line also allows for gene deletion during gland development (Kelleher et al. 2025), CXCL15 is expressed in the lung, adrenal glands, gastrointestinal and urogenital tracts, necessitating caution regarding extra‐uterine effects (Ohneda et al. 2000; Rossi et al. 1999; Schmitz et al. 2007). Similarly, off‐target recombination in non‐uterine tissues, such as the liver, skin, and oviductal epithelium, cannot be entirely avoided in our *Rdh1‐iCre* model (Figure S2). Careful consideration is required, as genetic alteration in these tissues could potentially affect the observed phenotype depending on the gene being targeted. Given the distinct expression profiles of the available Cre lines, the choice of a specific model should be carefully tailored to the experimental goals and the tissue‐specificity required.

In conclusion, the *Rdh1‐iCre* mouse is a versatile and potent tool for elucidating the development and physiological functions of uterine glands, as well as investigating the role of glandular abnormalities in various diseases, including endometrial cancer and adenomyosis.

## 4 Methods

### 4.1 Animals

All animal procedures were approved by the Ethical Committee for Vertebrate Experiments at Azabu University (ID#200312‐24). All experiments were conducted in accordance with the relevant guidelines and regulations, including the Animal Research: Reporting of In Vivo Experiments (ARRIVE) guidelines. *ROSA26‐H2B‐mCherry* mice <Stock No: CDB0204K> (https://large.riken.jp/distribution/reporter‐mouse.html, accessible as of April 2, 2026) were provided by RIKEN Center for Biosystems Dynamics Research (Abe et al. 2011). All mice were fed *ad libitum* under a controlled 12‐h light/12‐h dark photocycle at 23°C ± 2°C.

### 4.2 Targeting Construct

The targeting vector was constructed using genomic DNA extracted from mouse liver. Fragments flanking the stop codon of the *Rdh1* gene (the 5′ and 3′ homology arms) were amplified using PrimeSTAR Max DNA Polymerase (Takara Bio, Shiga, Japan) with specific primers incorporating AgeI and NheI restriction sites. The primer sequences were as follows: Rdh1‐upper forward: 5′‐ATACCCGGTAGGATCCAGCCGGGAGCTGGCCTGTGAG‐3′, Rdh1‐upper reverse: 5′‐CTGCTAGCGAGGGCTTTCTCAGGCTTCATGGAAGTC‐3′, Rdh1‐lower forward: 5′‐TTACCGGTCATCGCTAGCAGTGTTCACCTATGTGCATACCTGG‐3′, and Rdh1‐lower reverse: 5′‐TGCTGCTGGAGGAGTGTGGCAGTGG‐3′. The resulting amplicons were cloned into the pCR2.1‐TOPO vector using the Zero Blunt PCR Cloning Kit (Thermo Fisher Scientific, Waltham, MA, USA). Following restriction enzyme digestion, the 5′ and 3′ homology arms were ligated. Subsequently, a cassette consisting of T2A‐iCreS (codon‐improved Cre recombinase containing a splice intron from the SV40 T‐antigen gene) (Inoue et al. 2018), a WPRE (Woodchuck Hepatitis Virus Posttranscriptional Regulatory Element), and a polyA signal (rabbit β‐globin polyadenylation signal) was inserted into the NheI site using the In‐Fusion HD Cloning Kit (Takara Bio). The final targeting construct contained approximately 1.2 kb of 5′ homology and 1.1 kb of 3′ homology, designed for targeted insertion immediately upstream of the stop codon within the *Rdh1* locus (Figure 1A).

### 4.3 Generation of Rdh1‐iCre Mice

*Rdh1‐iCre* embryonic stem (ES) cell lines were established using CRISPR/Cas9‐mediated genome editing, as previously described (Ozawa et al. 2022). Briefly, 1.0 × 10^5^ V6.5 ES cells (C57BL/6 × 129SvJae) were transfected with 1 μL of the targeting construct and 1 μL of Cas9 ribonucleoprotein (RNP) complexes using the Neon NxT Electroporation System 10 μL Kit (Thermo Fisher Scientific). The RNP complex consisted of Alt‐R S.p. Cas9 Nuclease V3 (0.3 μg/μL; IDT, Coralville, IA, USA) and a guide RNA (gRNA; 10 μM; IDT: 5′‐CACATAGGTGAACACTTCAG‐3′), supplemented with Electroporation Enhancer (21.6 μM, IDT). Electroporation was performed with two pulses at 1200 V for 20 ms. Following electroporation, cells were cultured in 60 mm dishes and passaged once prior to colony selection. Correctly targeted clones resulting from homologous recombination were identified by PCR‐based genotyping using the following primers: Rdh1‐iCre 5′KI forward (KI 5F): 5′‐CAAGGCAGATGGAAGAGGTTGAGCCAG‐3′, Rdh1‐iCre 5′KI reverse (KI 5R): 5′‐TGGAGACTTTCCTCTTCTTCTTGGGCACC‐3′, Rdh1‐iCre 3’KI forward (KI 3F): 5′‐GGTGCAGGCTGCCTATCAGAAGGTG‐3′ and Rdh1‐iCre 3’KI reverse (KI 3R): 5′‐AGCCCAGGATTCTGTCTCTCCCACAC‐3′. Initial screening for successful knock‐in was performed by PCR, yielding a 1367 bp fragment (using primers KI 5F and KI 5R) and a 1764 bp fragment (using primers KI 3F and KI 3R) (Figure 1A,B). Furthermore, a 4846 bp amplicon was confirmed using the KI 5F and KI 3R primer combination, and the junctions between the homology arms and the knocked‐in cassette were verified at both ends by Sanger sequencing. Ultimately, 5 out of 23 clones (21.7%) were confirmed to harbor the correct knock‐in allele. Potential off‐target cleavage was not assessed. Confirmed ES cell lines (selected two clones) were injected into blastocysts derived from ICR mice (Japan SLC, Shizuoka, Japan), which were subsequently transplanted into pseudopregnant ICR females to generate chimeric offspring. Male chimeras were crossed with C57BL/6J females (Jackson Laboratory Japan, Kanagawa, Japan). The resulting F1 females were backcrossed with C57BL/6J males for at least six generations. Mice were maintained in the barrier facility at Azabu University. Routine genotyping was performed via PCR using tail genomic DNA and the following primers; Rdh1‐iCre forward (iCre F): 5′‐GAAGGACATATGGGAGGGCA‐3′, Rdh1‐iCre‐WT reverse (iCre‐WT R); 5′‐ACATCTCCCCAGGTATGCAC‐3′ and Rdh1‐WT forward (WT F): 5′‐CCACATAAAGGGAACCTGGCT‐3′. The iCre F and iCre‐WT R primers amplify a 363 bp band (knock‐in), while the WT F and iCre‐WT R primers amplify a 605 bp WT band (Figure 1A,C). To valid the Cre recombinase activity, *Rdh1‐iCre* males were crossed with *ROSA26‐H2B‐mCherry* (*flox*/*flox*) females, and their offspring were used for further analysis.

### 4.4 Fertility Test

Sexually matured male mice were caged with sexually matured female mice. Copulation was confirmed by checking for vaginal plugs every morning (0900–1100 h). Plugged females were separated and monitored during pregnancy. The number of pups was counted after parturition (20–21 days after plug confirmation).

### 4.5 RNA Extraction and Quantitative PCR (qPCR)

Total RNA was isolated from uterine and liver tissues using the RNeasy Mini Kit (Qiagen, Hilden, Germany) according to the manufacturer's instructions. The concentration of total RNA was measured using a NanoDrop system (ND‐1000, Thermo Fisher Scientific). One microgram of total RNA was reverse‐transcribed into cDNA using SuperScript III Reverse Transcriptase (Thermo Fisher Scientific) with oligo dT primer. Quantitative PCR (qPCR) was performed using a QuantStudio 5 Real‐Time PCR System (Thermo Fisher Scientific) with Power SYBR Green Master Mix (Thermo Fisher Scientific). The primer sequences used were as follows: *Rdh1*, forward 5′‐TTAGGAGGGTTGAGGGAGGG‐3′ and reverse 5′‐TGGACATCAGCCAGCACTTT‐3′; *Gapdh*, forward 5′‐AGGTCGGTGTGAACGGATTTG‐3′ and reverse 5′‐TGTAGACCATGTAGTTGAGGTCA‐3′. Relative expression levels of target transcripts were normalized to *Gapdh* and calculated using the ΔΔ*C*_t_ method (Livak and Schmittgen 2001). The mean value of the control samples was set to 1.0.

### 4.6 Histological Analysis

Fixed tissues in 4% paraformaldehyde were cryoprotected by sucrose infiltration and embedded in optimal cutting temperature (OCT) compound (Sakura Finetek Japan, Tokyo, Japan). Frozen sections (10 μm) were prepared, rehydrated in phosphate‐buffered saline (PBS), and mounted with ProLong Glass Antifade Mountant with NucBlue Stain (P36981, Thermo Fisher Scientific). Fluorescence images were acquired using a BZ‐X700 or BZ‐X800 microscope (Keyence, Osaka, Japan), and fluorescent signals derived from *ROSA26‐H2B‐mCherry* were examined.

### 4.7 Immunostaining

Immunostaining was performed as previously described (Namiki et al. 2023). Briefly, frozen sections were rehydrated in PBS. After blocking with 5% bovine serum albumin (BSA), the sections were incubated with primary antibodies (listed in Table 1) overnight at 4°C. The sections were then incubated with Alexa Fluor 488‐ or 647‐conjugated secondary antibodies (Jackson ImmunoResearch Laboratories, West Grove, PA, USA) for 1 h and mounted with ProLong Glass Antifade Mountant with NucBlue Stain. Images were acquired using a BZ‐X700 or BZ‐X800 (Keyence) microscope. All signals were detected under the same lighting conditions for the control and *Rdh1‐iCre* groups. Channel colors were assigned (pseudocolored) for visualization. mCherry‐ and/or FOXA2‐positive cells were counted in randomly selected sections, and the average percentage was calculated for each sample. Data are presented as mean ± standard deviation.

**TABLE 1:** Primary antibody list.

Target	Species	Source	Catalog no.	RRID	Dilution
KRT8	Rat	DSHB	TROMA‐I	AB_531826	1:50
FOXA2	Rabbit	Abcam	ab108422	AB_11157157	1:200
CYP17A1	Rabbit	CST	#94004	AB_2800219	1:200
VIM	Mouse	DAKO	M7020	AB_2304493	1:200

### 4.8 Statistical Analysis

Statistical analyses were performed using R (ver. R4.4.1). Data are presented as the mean ± standard error of the mean (SEM). Comparisons between two groups were conducted using Student's *t*‐test or Werch *t*‐test, and *p* < 0.05 was considered statistically significant.

## Funding

This work was supported by Japan Society for the Promotion of Science, JP21K09512, JP24K01950, JP25KJ2187, JP22H04922, and JP25K22429 and Azabu University.

## Supporting information


**Figure S1:** Representative fluorescence images of the neonatal uterus (1 week of age). A weak mCherry signal (red, arrowhead) was detected in only a few epithelial cells. Scale bar = 100 μm.
**Figure S2:** Endogenous mCherry (red) expression in the liver and skin of *Rdh1‐iCre* reporter mice. Scale bars = 100 μm.

**Data S1:** Technology Report Checklist.

## Data Availability

All data generated or analyzed during this study are available from the corresponding author upon reasonable request.
