# Supplementary material for: Generation and Characterization of a Rdh1‐iCre Line to Study Uterine Glandular Biology
Source: Genesis. 2026 Jul 23;64(4):e70068. doi: 10.1002/dvg.70068 (PMC13396816; doi:10.1002/dvg.70068)
Supplement: Supplementary file 1 — Figure S1: Representative fluorescence images of the neonatal uterus (1 week of age). A weak mCherry signal (red, arrowhead) was detected in only a few epithelial cells. Scale bar = 100 μm. Figure S2: Endogenous mCherry (red) expression in the liver and skin of Rdh1‐iCre reporter mice. Scale bars = 100 μm. [file DVG-64-e70068-s001.docx]

**
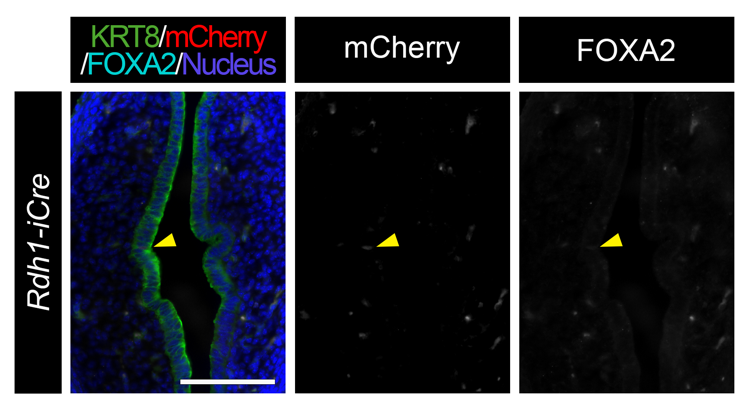
**

**Supplemental figure 1**

Representative fluorescence images of the neonatal uterus (1 week of age). A weak mCherry signal (red, arrowhead) was detected in only a few epithelial cells. Scale bar = 100 μm.

**
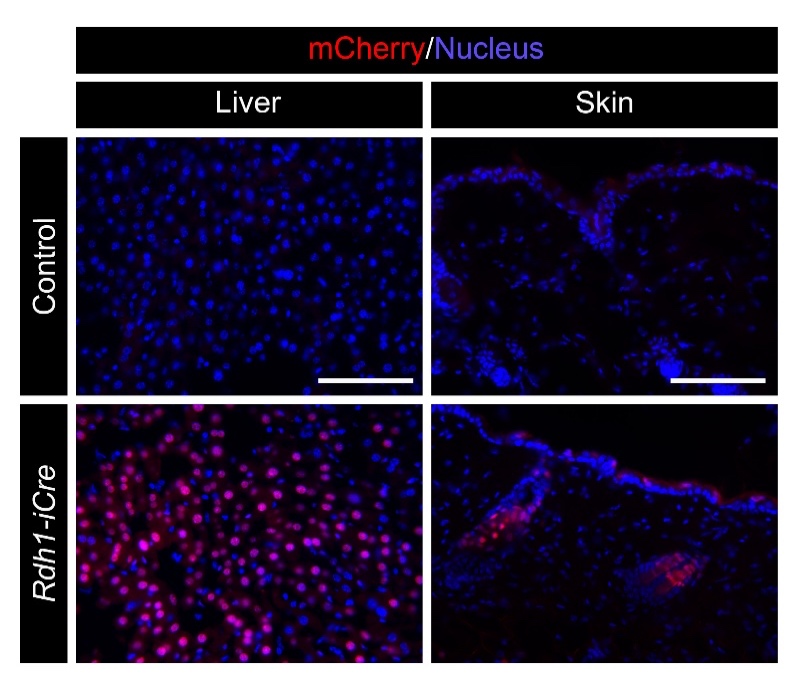
**

**Supplemental figure 2**

Endogenous mCherry (red) expression in the liver and skin of *Rdh1-iCre* reporter mice. Scale bars = 100 μm.
