# Supplementary material for: Generation and Characterization of a Rdh1‐iCre Line to Study Uterine Glandular Biology
Source: Genesis. 2026 Jul 23;64(4):e70068. doi: 10.1002/dvg.70068 (PMC13396816; doi:10.1002/dvg.70068)
Supplement: Supplementary file 2 — Data S1: Technology Report Checklist. [file DVG-64-e70068-s002.pdf]

**Technology Report Checklist**	**Yes**	**No**	**N/A**
* Does the technology or reagent provide an advance in addressing a developmental problem or process?	◉	○	○
* If a transgenic line is reported, do you describe how this line is distinct from other published lines?	○	○	◉
* Is the developmental relevance of the technology/reagent experimentally demonstrated? This includes showing the expression of a reporter at various stages, including both whole mount and tissue section analyses. The demonstration that a ubiquitous Cre-deletion results in a phenotype already reported for a gene knock-out will not be sufficient.	◉	○	○
* For new Cre-driver lines, do you provide proof of activity?	◉	○	○
* For new Cre-driver lines, do you provide evidence concerning the specificity and penetrance of the effects (i.e. weak or strong and whether activity is mosaic) and the length of time/stage before Cre activity is detected?	◉	○	○
* For transgenic lines, do you show whether there are any phenotype(s) associated with homozygosity for the transgene insertion?	○	○	◉
* If a reagent or transgenic animal is described, have you declared in the manuscript in the Methods section that it will be available to the research community upon acceptance of the manuscript?	○	○	◉
